# The Consistent Tick-Vertebrate Infectious Cycle of the Lyme Disease Spirochete Enables Borrelia burgdorferi To Control Protein Expression by Monitoring Its Physiological Status

**DOI:** 10.1128/jb.00606-21

**Published:** 2022-04-05

**Authors:** Brian Stevenson, Andrew C. Krusenstjerna, Tatiana N. Castro-Padovani, Christina R. Savage, Brandon L. Jutras, Timothy C. Saylor

**Affiliations:** a Department of Microbiology, Immunology, and Molecular Genetics, University of Kentucky, Lexington, Kentucky, USA; b Department of Entomology, University of Kentucky, Lexington, Kentucky, USA; c Department of Biochemistry, Virginia Tech, Blacksburg, Virginia, USA; University of Illinois at Chicago

**Keywords:** *Borrelia*, spirochetes

## Abstract

The Lyme disease spirochete, Borrelia burgdorferi, persists in nature by alternatingly cycling between ticks and vertebrates. During each stage of the infectious cycle, B. burgdorferi produces surface proteins that are necessary for interactions with the tick or vertebrate tissues it encounters while also repressing the synthesis of unnecessary proteins. Among these are the Erp surface proteins, which are produced during vertebrate infection for interactions with host plasmin, laminin, glycosaminoglycans, and components of the complement system. Erp proteins are not expressed during tick colonization but are induced when the tick begins to ingest blood from a vertebrate host, a time when the bacteria undergo rapid growth and division. Using the *erp* genes as a model of borrelial gene regulation, our research group has identified three novel DNA-binding proteins that interact with DNA to control *erp* transcription. At least two of those regulators are, in turn, affected by DnaA, the master regulator of chromosome replication. Our data indicate that B. burgdorferi has evolved to detect the change from slow to rapid replication during tick feeding as a signal to begin expression of Erp and other vertebrate-specific proteins. The majority of other known regulatory factors of B. burgdorferi also respond to metabolic cues. These observations lead to a model in which the Lyme spirochete recognizes unique environmental conditions encountered during the infectious cycle to “know” where they are and adapt accordingly.

## INTRODUCTION

All organisms require mechanisms to detect their environment, in order to produce only those proteins that are useful in specific conditions. This is particularly important for vector-borne pathogens, which must survive in two distinct host types while also facilitating transmission back and forth between those hosts. An understanding of a pathogen’s sensory and signal transduction mechanisms may reveal critical components that can be targeted for development of novel therapies.

Lyme disease (Lyme borreliosis) is a potentially debilitating disease of humans and domestic animals. The incidence of this disease has been steadily increasing, in part due to expanding ranges of vector ticks (1). Lyme disease can manifest in many tissues and organs, with symptoms that include arthritis, meningitis, facial nerve paralysis, and atrioventricular nodal block. Failure to treat this infection promptly and adequately can result in persistent debilitating effects or, sometimes, death (2–4).

The infectious agent of Lyme disease, Borrelia burgdorferi *sensu lato* (hereafter called B. burgdorferi, for simplicity), does not produce classical toxins or other recognizable virulence factors that directly cause host damage. Instead, disease symptoms are due to overly robust immune system reactions to bacterial components (5–10). Thus, to cause clinical disease, B. burgdorferi needs only to colonize and survive in the patient’s body.

B. burgdorferi is transmitted to humans and other vertebrates through the bites of infected *Ixodes* species ticks. Transmission is a complex, multistep process (5, 11, 12). First, bacteria within the midgut of a tick detect when the tick vector is imbibing a blood meal, change from a relatively inert metabolism to one of rapid replication and movement, and then alter the composition of their outer membrane proteins from tick-specific adhesins to proteins that are optimal for vertebrate infection. Next, bacteria penetrate the tick midgut wall, invade the salivary glands and ducts, and pass with saliva into the bite wound. Following inoculation into the vertebrate host, B. burgdorferi migrates through solid host tissues and in the bloodstream and then establishes long-term colonization of a variety of organs and tissues. These complexities require that B. burgdorferi efficiently determines its location in the tick-vertebrate infectious cycle and produces only those proteins and other factors that are essential for that time and place.

The Lyme disease spirochete encodes only two regulatory two-component systems (13, 14). Only one of those, Hk1-Rrp1, includes a signal receptor/histidine kinase that passes through the inner membrane and can, therefore, detect an external signal(s) (15–19). The other two-component system, Hk2-Rrp2, has a cytoplasmic receptor/histidine kinase, implying response to an internal signal(s) (19–25). However, investigations have revealed that B. burgdorferi employs a complex signaling network to control production of proteins (26–28). With the exception of Hk1-Rrp1, all known regulatory factors are controlled by rates of bacterial replication and/or levels of cytoplasmic factors (27–29). That realization begs the question of why B. burgdorferi can coordinate differential expression of numerous proteins during its complex infectious cycle by sensing internal cues.

Herein, we describe the elucidation of signaling pathways that are used by B. burgdorferi to control production of a family of infection-associated proteins, along with a hypothesis of how that network meshes with the Lyme spirochete’s natural tick-vertebrate infectious cycle.

## B. BURGDORFERI AS A MODEL VECTOR-BORNE PATHOGEN

In addition to being the causative agent of a significant human disease, B. burgdorferi has emerged as a valuable model organism for studies of vector-borne bacteria and of the *Spirochaetota* phylum in general. The following features of B. burgdorferi contributed to completion of the studies described in the remainder of this review and which continue to advance our understanding of this infectious bacterium and the pathogenesis of Lyme disease.

B. burgdorferi is readily cultivated in the laboratory (30, 31). Although currently used culture media are complex and contain undefined ingredients, analyses of bacterial physiological responses can be readily observed in response to variations in concentrations of medium ingredients, temperature, pH, osmotic strength, or other factors (19, 29, 32–34). For instance, replacement of glucose with other compounds enabled characterization of carbohydrate utilization by B. burgdorferi (35).

Additionally, the entire infectious cycle of the Lyme disease bacterium can be replicated in the laboratory, using ticks and model vertebrate hosts such as mice, rats, rabbits, and macaques (36–39). Thus, functions for proteins and other bacterial factors can be precisely assessed during infection processes.

B. burgdorferi is a member of *Spirochaetota*, a phylum of bacteria that diverged many millennia ago from the ancestors of the more commonly studied *Proteobacteria* and *Firmicutes* (40, 41). Initial attempts to genetically manipulate B. burgdorferi were unsuccessful, due to a combination of commonly used selectable markers not being transcribed by the spirochete and the inability of commonly used plasmids to replicate (42, 43). However, a stepwise approach, from recombination of an antibiotic resistance-encoding mutation into the chromosome, followed by use of that locus to integrate circular DNA via single crossover, led to the development of numerous selectable markers, cloning vectors, fluorescent tags, and other genetic tools (42–60). CRISPR-interference (CRISPRi) methods were recently developed and have been exploited to knock down expression levels of B. burgdorferi proteins (61).

B. burgdorferi naturally contains multiple, distinct replicons, some of which encode essential infection-associated genes, and extended cultivation may result in spontaneous loss of one or more of those replicons (13, 14, 62–64). However, there are simple methods to screen transformants for the presence of all replicons, adding a step to the generation of mutants in infectious bacteria but a step that is usually not insurmountable (65). Studies of cultured B. burgdorferi can be simplified by using clonal strains with minimal, yet highly stable, genomes (66, 67). To date, all known regulatory factors of Lyme disease *Borrelia* spp. are encoded on replicons that are never, or rarely, lost during cultivation (27, 28).

## INITIAL OBSERVATIONS OF GENE/PROTEIN REGULATION AND SUBSEQUENT MODELS

**FIG 1:**
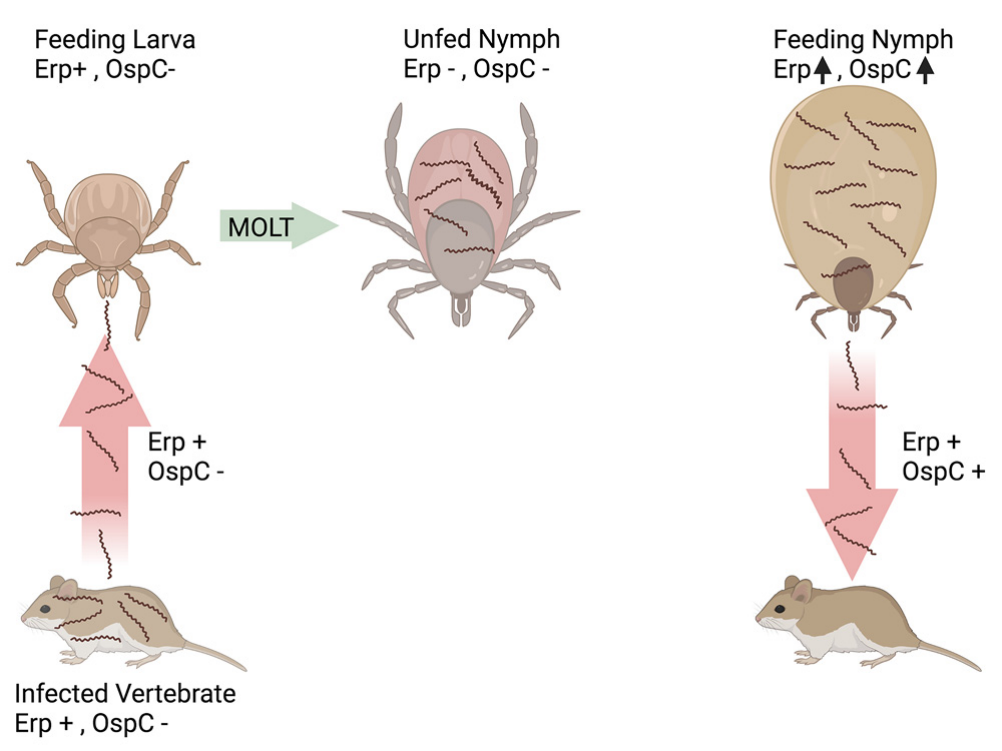
Natural infectious cycle of the Lyme disease spirochete. There are three postembryonic life stages of *Ixodes* species ticks: larva, nymph, and adult (not shown). Each stage feeds once and then molts to the next stage. Adult females feed once, lay eggs, and then die. In areas where Lyme disease is endemic, larvae and nymphs feed on the same vertebrate species, while adults feed on different hosts and therefore do not play major roles in the maintenance of B. burgdorferi. There is essentially no transmission of B. burgdorferi through tick eggs, so larvae are uninfected upon hatching. Larvae acquire B. burgdorferi if they feed on an infected reservoir vertebrate. Bacteria colonize the tick’s midgut and persist through the molt into the nymph stage. When a nymphal tick begins to feed, bacteria in the midgut undergo significant metabolic changes, penetrate the midgut wall, enter the salivary glands, and are injected into the vertebrate host with the tick’s saliva. B. burgdorferi in persistently infected vertebrates produces Erp surface proteins but not OspC. The spirochetes cease production of Erps after acquisition by feeding larvae, such that unfed nymphal ticks are colonized by bacteria that lack both Erps and OspC. When the tick begins to feed, OspC and Erps are induced, and transmitted bacteria produce both classes of surface proteins. B. burgdorferi spirochetes cease production of OspC within a few days of vertebrate infection but continue to express Erps on their outer surfaces.

Despite the infectious agent of Lyme disease having been identified in 1982 (68, 69), the first investigations of gene and protein regulation by B. burgdorferi were not published until 1995. B. burgdorferi requires a specific outer surface protein, OspC, for the initial stages of vertebrate infection (70–81). In their seminal studies, Schwan and colleagues observed that B. burgdorferi within the midguts of unfed ticks did not produce OspC, while the onset of tick feeding induced the bacteria to produce substantial quantities of OspC (32) (Fig. 1).

Schwan et al. further observed that B. burgdorferi cultured at 23°C produced significantly less OspC protein than did bacteria that had been transferred from 23 to 37°C (32). This change is not a heat shock response but requires several generations of bacterial replication at the warmer temperature (33). Such temperature shift studies are generally accomplished in the laboratory by diluting a mid-exponential phase 23°C culture 1:100 into fresh medium, incubating the new aliquot at 35 to 37°C to mid-exponential phase, and then harvesting both cultures at the same time (32, 33). Simultaneous harvesting is possible because B. burgdorferi replicates several times faster when cultured at 35 to 37°C than it does at 23°C (33).

The choices of 23°C and 35 to 37°C were made because they mimic temperatures experienced by B. burgdorferi in unfed and feeding ticks, respectively (32). Some other vector-borne bacteria are known to modulate their proteomic profiles in response to temperature (82, 83). Those results led to hypotheses that B. burgdorferi possesses mechanisms to sense temperature, which then conveys a signal(s) to affect transcription of target genes. To date, a thermosensitive regulator has not been experimentally demonstrated to exist in B. burgdorferi.

Subsequent studies revealed that it is actually the change in bacterial replication rate that results in changed levels of OspC and several other surface proteins (29). Moreover, differences in replication rates also affected levels of the regulatory proteins known to control *ospC* and other loci. This was demonstrated through the use of two incomplete formulations of culture medium that support B. burgdorferi replication at 35°C at rates that are similar to growth in complete medium at 23°C (29). B. burgdorferi was then cultured to mid-exponential phase in either of the incomplete media at 35°C and then diluted into complete medium and cultured to mid-exponential phase at 35°C. All of the slow-replication conditions (either of the incomplete media at 35°C or complete medium at 23°C) resulted in substantially lower levels of OspC protein than after passage to the fast-replication condition (complete medium at 35°C) (29). Further indication that temperature, *per se*, was not the signal for OspC repression/induction was observed when B. burgdorferi that had been stored at −80°C was inoculated into complete medium and grown at 23°C. Bacteria passaged from −80 to 23°C produced OspC at levels comparable to those normally observed after passage from 23°C to 35 to 37°C (29). Thus, the condition before passage to 23°C impacted the expression of OspC. Note that a mechanism that strictly detects temperature would not be affected by the preceding conditions. The significance of B. burgdorferi’s ability to detect changes in replication rate and respond by altering its proteome are discussed further below.

Since the initial observations of Schwan et al. in 1995 (32), numerous studies have been undertaken to elucidate mechanisms by which the Lyme disease spirochete senses its environment and controls expression of genes and proteins. While genetic and biochemical analyses have identified several DNA-binding proteins and other factors that have impacts on gene expression (26–28), few published studies have examined their mechanisms of action at a molecular level. To date, the most completely defined mechanism is that which controls production of B. burgdorferi Erp proteins. Analyses of that gene and protein family have provided important insights on borrelial sensory and regulatory mechanisms, as follows.

## B. BURGDORFERI *erp* GENES AND Erp PROTEINS

Individual Lyme disease spirochete cells naturally maintain numerous *erp* operons, each of which is carried on an episomal cp32 prophage (14, 64, 84–92). The original isolate of the B. burgdorferi type strain, B31, contained at least 11 cp32-derived episomes, 10 of which carry mono- or bicistronic *erp* operons, and encoded 13 distinct Erp outer surface lipoproteins (13, 14, 85, 86). Some borrelial *erp* genes have been given various other names, including *ospE*, *ospF*, *elp*, *bbk2.10*, *bbk2.11*, and *p21*, but we consider it easier to view them as a single family of genes, since all possess highly conserved features such as (i) genomic location, (ii) promoter and operator DNA sequences, and (iii) regulatory mechanisms and (iv) encode surface-localized lipoproteins with conserved leader and N-terminal amino acid sequences (91, 93).

All Erp proteins are surface-exposed lipoproteins (94, 95). Dual-labeling studies found that all of the Erp proteins of B. burgdorferi type strain B31 are simultaneously coexpressed (96). Immunofluorescence microscopy and quantitative reverse transcription (RT)-PCR determined that B. burgdorferi produces little to no detectable amounts of any Erp protein during colonization of unfed tick midguts, but the genes and proteins are induced upon tick feeding (97, 98). The *erp* genes and Erp proteins are expressed during transmission from tick to vertebrates, throughout vertebrate infection, and continue to be expressed during transmission from infected vertebrates to naive ticks (Fig. 1) (98, 99). Consistent with that expression profile, all identified functions of Erp proteins involve interactions between B. burgdorferi and its vertebrate hosts, including binding to complement factors H, C1r, and C1s, plasmin, laminin, and glycosaminoglycans (100–104).

As noted above, almost all Lyme disease borreliae naturally contain numerous *erp* operons, each located on a separate cp32 prophage. Why do individual bacteria contain multiple, distinct *erp* genes? One early hypothesis suggested that *erp* loci might recombine during vertebrate infection as a mechanism to generate antigenic diversity. However, analyses of clonal strains before, during, and after passage through mice revealed absolute conservation of *erp* sequences (105, 106). A report suggesting genetic variation was later found to have undoubtedly detected PCR artifacts (106, 107). At present, the most reasonable explanation for the simultaneous coexpression of numerous Erp proteins with distinct sequences is due to the broad host range of vector ticks. For example, Ixodes scapularis in the northeastern United States might feed on rodents, birds, or other vertebrates (108). Only the nymph and adult stages of *Ixodes* species ticks can transmit Lyme disease spirochetes, and each stage feeds only once (Fig. 1). For B. burgdorferi to successfully infect a vertebrate, the bacteria must be able to interact with the new host’s tissues while also fending off immune system responses. Those features often differ considerably between the various potential hosts of B. burgdorferi. Continuing our example, B. burgdorferi in an I. scapularis tick in the northeastern United States cannot “predict” the type of host that the tick is feeding on, and so Lyme spirochetes express a wide variety of host-interactive surface proteins during transmission to increase the probability that their surface proteomes are adequate to infect the vertebrate into which they are deposited. Supporting this hypothesis, different Erp proteins exhibit different affinities for the complement factor H proteins of different potential vertebrate hosts (109, 110). This hypothesis can also explain why B. burgdorferi simultaneously produces numerous other polymorphic protein families, such as the CspA/pFam54, Rev, and Mlp outer surface proteins (111–118).

## MECHANISMS CONTROLLING TRANSCRIPTION OF *erp* OPERONS

DNA sequences 5′ of all *erp* operons are highly conserved, and a single transcriptional start site was mapped to 14 bp 5′ of the initial *erp* gene’s start codon (66, 119). To identify *cis* sequences required for regulation of *erp* transcription, *erp* 5′ DNA sequences were fused to *gfp*, and then independently replicating plasmids with each construct were introduced into B. burgdorferi (66). As noted above, *erp* operons are transcribed at greater levels when B. burgdorferi are cultured at 35 to 37°C than at 23°C. Thus, transformants were cultured at both temperatures, and bacterial green fluorescent protein (GFP) levels were determined by flow cytometry (66). Successive deletions of 5′ DNA revealed that an approximately 145-bp region 5′ of the transcriptional start site is necessary for regulation of transcription. Deletion of that region resulted in constitutive, high-level transcription (66). This regulatory region was designated the *erp* operator.

Electrophoretic mobility shift assays (EMSAs) with B. burgdorferi cytoplasmic extracts detected that protein(s) bound specifically to *erp* operator DNA (66). DNA affinity chromatography/pulldown was then employed to purify proteins that bound to the *erp* operator (120, 121). Three proteins were detected, which were subsequently identified by mass spectrometry. All three are novel nucleic acid-binding proteins: BpaB (borrelial ParB analog) (122), BpuR (borrelial PUR domain) (123), and EbfC (*erp*-binding factor, chromosomal) (120).

All small borrelial replicons, including the cp32s, carry a *bpaB* gene. In most replicons, *bpaB* is located 3′ of a putative *parA*. Biochemical studies indicate that BpaB is analogous to the ParB proteins of other bacterial replicons. Each B. burgdorferi replicon contains a unique *parA-bpaB* locus, which is presumed to enable the compatibility of multiple replicons within individual cells (13, 14, 64, 86, 124). However, the various *parA-bpaB* pairs are maintained across different isolates. For example, 12 distinct *parA-bpaB* pairs have been identified in the cp32 prophages of Lyme disease spirochete isolates collected throughout the world (64, 91, 124, 125). All cp32-encoded BpaB proteins contain a conserved amino acid sequence that is not found in any other type of BpaB, and that domain is required for binding to *erp* operator DNA (122). As a consequence, BpaB proteins from any cp32 will bind to the *erp* operator elements of all of the *erp* loci in that bacterial cell (122). This cross talk would facilitate the observed simultaneous coexpression of all *erp* operons in a bacterium (96).

**FIG 2:**
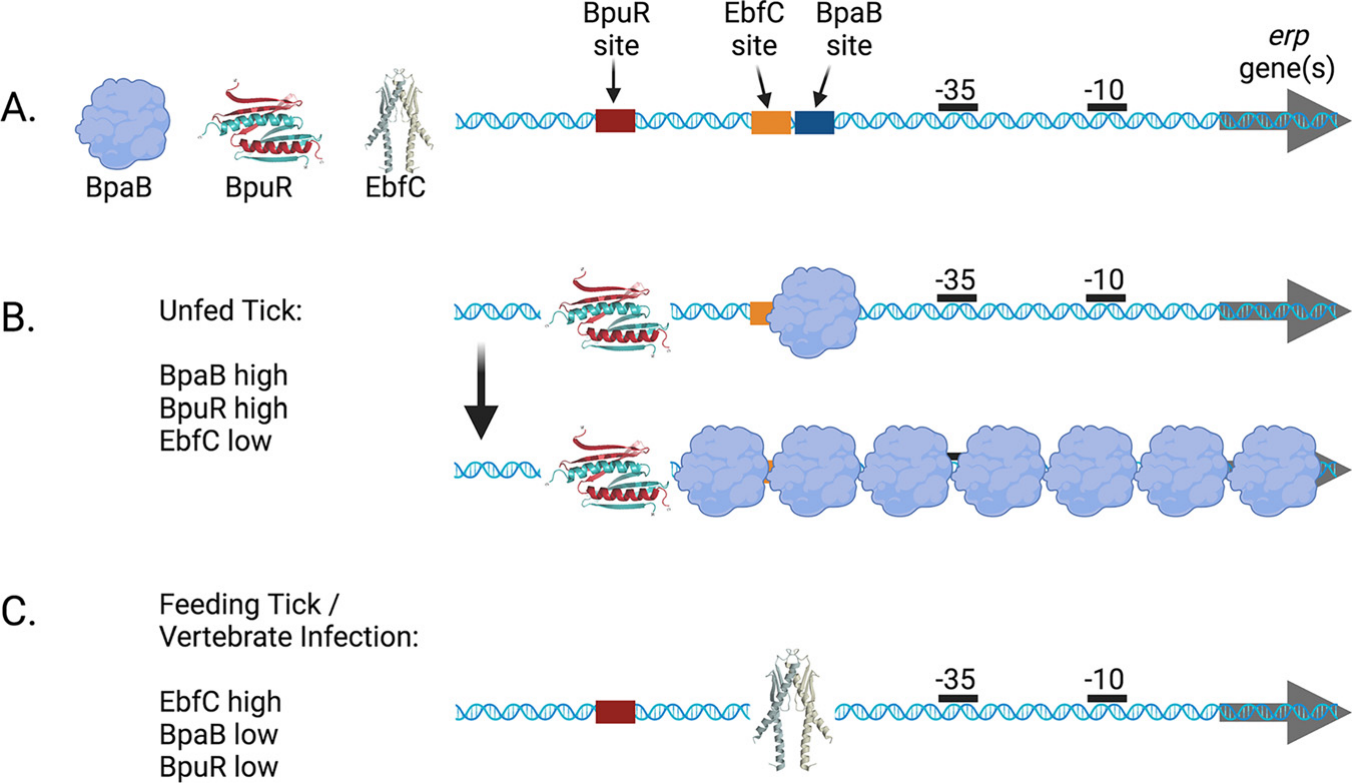
Mechanism by which B. burgdorferi controls transcription of *erp* operons. (A) All *erp* operons include a 5′ operator that consists of a BpaB-binding site, two to three EbfC-binding sites, and a BpuR-binding site. The BpaB- and EbfC-binding sites are adjacent, and the two proteins compete for binding to the DNA. (B) In the midgut of an unfed tick, or under slow-replication culture conditions, BpaB and BpuR levels are elevated, while EbfC levels are low. BpaB binds to its high-affinity binding site and then spreads along the DNA, stabilized by protein-protein interactions. The presence of BpuR enhances transcription repression by BpaB, possibly by influencing the direction of BpaB multimerization. (C) In a feeding tick, during vertebrate infection, or during culture under rapid replication conditions, EbfC levels are high, while BpaB and BpuR levels are low. Binding of EbfC to *erp* operator DNA blocks binding by BpaB, freeing the promoter for recognition by RNA polymerase. Promoter −35 and −10 elements are indicated by solid black bars 5′ of the open reading frames (ORFs). The illustrated structures of BpuR and EbfC are adapted from the solved and modeled proteins (123, 128, 131).

BpaB serves as the repressor of *erp* transcription. A high-affinity BpaB-binding site is located 5′ of all *erp* promoter elements (Fig. 2). BpaB bound to this site facilitates binding of additional BpaB proteins to adjacent DNA. The lower affinity for those DNA sequences is apparently offset by protein-protein interactions between the BpaB molecules. BpaB spreads along DNA from the initial nucleation site and evidently prevents RNA polymerase from interacting with *erp* promoter sequences (122, 126). Consistent with BpaB being the *erp* repressor, cp32 *bpaB* genes are transcribed at higher levels during tick colonization than they are during vertebrate infection (126).

BpuR is encoded on the B. burgdorferi main chromosome and so is considered to be a host protein that is coopted by cp32 prophages for regulation of their own genes (120, 123). The *erp* operator elements contain a single BpuR-binding site, 5′ of the BpaB-binding sequence (Fig. 2). BpuR binding to that site enhances the repressive activity of BpaB, and it thus serves as a corepressor (123). Correlating with that activity, *bpuR* is expressed at greater levels in unfed ticks than it is during vertebrate infection (127).

**FIG 3:**
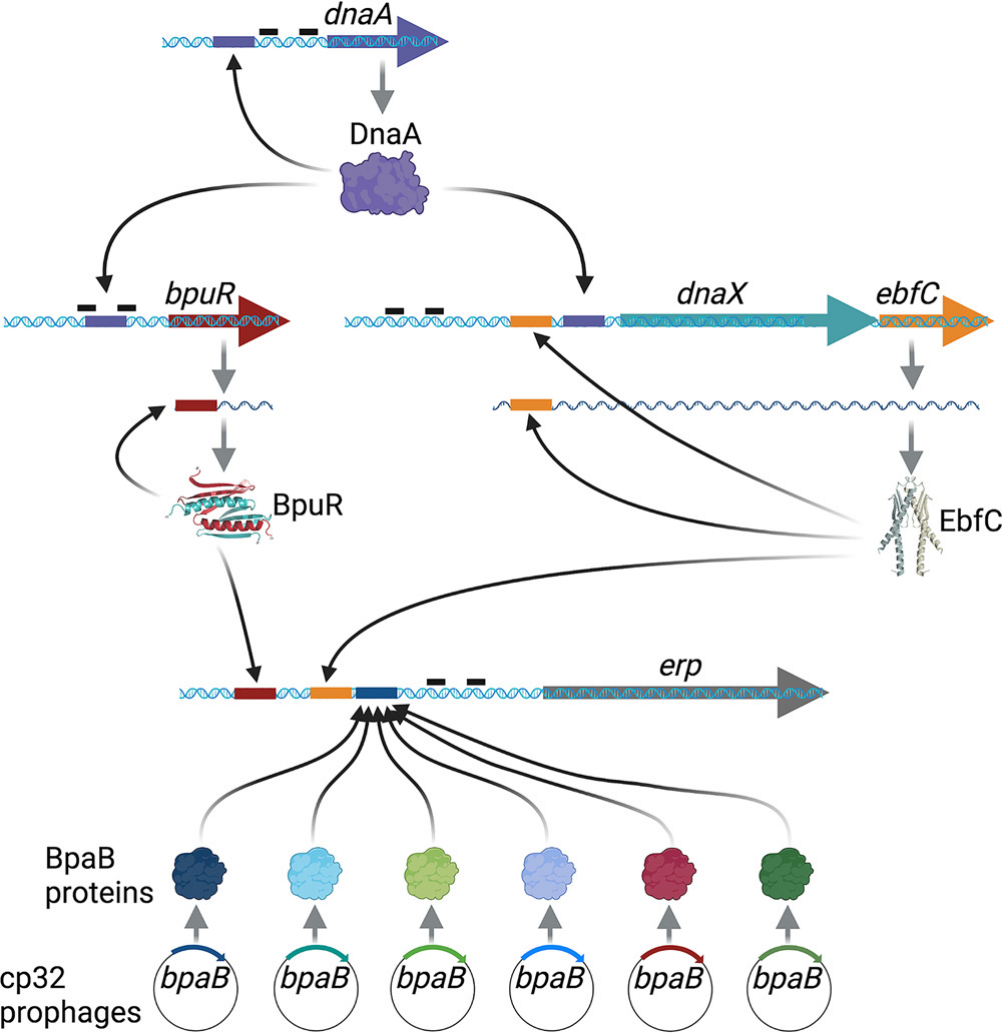
Current understanding of *erp* regulatory circuits. Each cp32 produces a distinct BpaB. However, all cp32-encoded BpaB proteins contain a conserved amino acid motif that binds to *erp* operator DNA. That feature permits cross talk and coordinated repression/derepression of all *erp* operons in a bacterium. BpuR can bind its own mRNA and inhibit translation. DnaA binds to *bpuR* promoter DNA between the −10 and −35 sequences. EbfC is cotranscribed with *dnaX*, which encodes the tau subunit of DNA polymerase III. Both DnaA and EbfC bind to sequences between the *dnaX-ebfC* promoter and the initiation codon of *dnaX*. EbfC also binds to the 5′ untranslated region of the *dnaX-ebfC* mRNA. DnaA binds to its own 5′ DNA. Promoter −35 and −10 elements are indicated by solid black bars 5′ of the ORFs. The illustrated structures of BpuR and EbfC are adapted from the solved and modeled proteins (123, 128, 131).

Homologs of BpuR are encoded by some other species of bacteria and, intriguingly, by all multicellular eukaryotes (123, 128, 129). The hallmark of these proteins is a structurally conserved “PUR” domain. All assessed PUR proteins bind to double-stranded DNA, single-stranded DNA, and RNA. Identified RNA targets of BpuR include its own mRNA, creating a feedback loop in which BpuR represses its own translation (Fig. 3) (127, 130). Additional binding sites have been detected throughout the borrelial transcriptome, and numerous physiological effects are apparent when BpuR is dysregulated (127).

EbfC is also encoded on the main chromosome (120). All *erp* operons contain 2 to 3 EbfC binding sites adjacent to their BpaB-binding site (Fig. 2). EbfC and BpaB compete for binding to the *erp* operator (131). EbfC can inhibit the transcriptional repression of BpaB and thus serves as the antirepressor (126). The *ebfC* gene is more highly transcribed during vertebrate infection than during tick colonization, paralleling the expression pattern of the *erp* genes (126).

Almost all known species of eubacteria encode a well-conserved homolog of EbfC, which has also been referred to as “ORF-12” or “YbaB” (132–137). We and others subsequently examined several EbfC homologs and determined that they, too, bind DNA (134, 135). Through studies that included visualization of GFP-tagged EbfC in B. burgdorferi, we determined that EbfC colocalizes with borrelial chromatin and fits the criteria of being a bacterial nucleoid-associated protein (“histone-like” protein) (138).

The mechanisms by which BpaB, BpuR, and EbfC control transcription of *erp* operons is diagrammed in Fig. 2. During colonization of the tick midgut, BpaB and BpuR are relatively highly expressed, while EbfC is repressed. As a result, BpaB binds to the high-affinity site in the *erp* operator and spreads along the DNA, enhanced by BpuR. When the tick begins to feed on a vertebrate host, BpaB and BpuR levels decrease, while EbfC is induced, resulting in EbfC outcompeting BpaB for the *erp* operator and facilitating promoter recognition by RNA polymerase.

## DnaA BINDS TO THE PROMOTER REGIONS OF THE *bpuR* AND *ebfC* OPERONS

Subsequent studies of BpaB, BpuR, and EbfC found that each regulates transcription and/or translation of numerous other genes and proteins (127, 138, 139). The majority of investigations have focused on the chromosomally encoded BpuR and EbfC proteins, since they control bacterial genes in addition to being coopted by the cp32 prophages for *erp* regulation.

The *bpuR* gene is located in a monocistronic operon (Fig. 3) (13, 123). The master regulator of chromosomal replication, DnaA, binds over the *bpuR* promoter (Fig. 3) (127). The apparent involvement of DnaA in the expression of *bpuR* is hypothesized to explain how BpuR concentration is coordinated with the rate of bacterial replication.

In B. burgdorferi, *ebfC* is the second gene in a bicistronic operon, following *dnaX*. The *dnaX* gene encodes the tau (τ) subunit of the DNA polymerase III holoenzyme. Both DnaA and EbfC proteins bind specifically to sites between the promoter and the *dnaX* start codon (A. C. Krusenstjerna, T.C. Saylor and B. Stevenson, unpublished data). As with the *bpuR* operon, binding of DnaA to the *dnaX-ebfC* operon may connect EbfC levels with the rate of bacterial replication. Involvement of DnaA in the expression of tau would also coordinate synthesis of the DNA polymerase III holoenzyme with DNA replication. Nearly all eubacteria encode a homolog of EbfC, and the majority also cotranscribe *ebfC* with *dnaX* (138). Little is known about how those other species regulate transcription of their *dnaX-ebfC* operons, other than there being evidence of a protein-binding site 5′ of *dnaX* in Escherichia coli and Caulobacter crescentus (140–143). Thus, detailed studies of the B. burgdorferi *dnaX-ebfC* operon may provide insight into a wide range of bacteria.

## OTHER REGULATED GENES AND PROTEINS

Other than the *erp* operons, the B. burgdorferi *ospC* and *rpoS* operons are the best studied, but our understanding of their regulatory mechanisms is far from complete. Both *rpoS* and *ospC* are repressed during colonization of unfed ticks and are greatly enhanced upon tick feeding (32, 144). Both genes are also influenced by the bacterial division rate in culture, their levels increasing upon shifts to conditions of rapid division (29). The effects of replication rate in culture parallel the effects on *erp* operons, as was described above.

Regulation of the *ospC* gene requires the RpoS alternative sigma factor (145, 146). Yet, other factors are undoubtedly at play with regulation of *ospC*, since that gene is repressed early during vertebrate infection while RpoS continues to be produced (147). A DNA sequence(s) 5′ of the *ospC* promoter is necessary for transcriptional regulation (148–150). The role(s) of that upstream sequence has yet to be fully examined.

RpoS plays critical roles in adapting B. burgdorferi for transmission from tick to vertebrate and for vertebrate infection (151). The *rpoS* gene is induced during tick feeding, but the molecular mechanisms underlying control of RpoS synthesis have yet to be defined. Another alternative sigma, RpoN, is necessary for *rpoS* induction, as is the activated form of the Rrp2 unit of the Hk2-Rrp2 two-component regulatory system (21–23, 145, 152–154). But at least one additional promoter is involved with transcription of *rpoS*, which is dependent upon the housekeeping sigma RpoD, and that transcript is influenced by the small RNA DsrA and the chaperone Hfq (155, 156). Two proteins, BosR and BadR, bind DNA sequences 5′ of *rpoS*, but their modes of action have not been fully elucidated (157–164). Another protein, BBD18, appears to affect RpoS through a posttranscriptional mechanism (165–168).

Despite uncertainties about the mechanisms controlling OspC and RpoS, it is clear that both are upregulated during tick feeding and in culture under conditions of rapid bacterial replication (29, 32, 147, 151, 169).

## METABOLIC CUES DIRECT B. BURGDORFERI PROTEIN PRODUCTION

It is important to recognize that even though the tick-vertebrate infectious cycle involves a large number of distinct interactions between B. burgdorferi and its hosts, the cycle never varies. The bacteria move from a feeding tick into a vertebrate, and then from that vertebrate into another tick, in a back-and-forth dance that has persisted for millennia. The absence of variation means that B. burgdorferi does not require the contingency systems that are found in free-living bacteria. Moreover, its predictable routine has evidently permitted B. burgdorferi to refine its regulatory system to detect conditions that occur only at specific times.

After B. burgdorferi is acquired by a feeding tick larva, and the tick completes digestion of that blood meal, the spirochetes enter an extended period of limited nutritional resources. Quantification of B. burgdorferi in tick midguts shows essentially no bacterial replication after the blood meal has been digested (12, 170). This period of low metabolic activity may last for many months. The tick’s next blood meal brings a rapid and dramatic change to the Lyme spirochete’s metabolism, with a doubling time of approximately 2 h (12, 170–173). The only time during B. burgdorferi’s infectious cycle where bacterial metabolism quickly shifts from slow to rapid is when the tick feeds on a vertebrate.

In the laboratory, shifting B. burgdorferi from a condition of slow replication to a condition of rapid replication mimics conditions in a tick as it initiates feeding and results in significant increases of Erp, EbfC, RpoS, and OspC proteins (29). Under those same conditions, levels of BpaB and BpuR decrease significantly (29). The evident roles of DnaA in controlling EbfC and BpuR, and ultimately Erp, link chromosomal replication and bacterial division to production of surface proteins that are involved with vertebrate infection. Continued bacterial replication during vertebrate infection would maintain the signals to produce Erp proteins.

We further hypothesize that other signals that affect borrelial gene regulation are also tied to the bacterium’s invariant life cycle (29, 147). Among known environmental signals are pH, osmolarity, carbon sources, redox potential, metal ions, and carbon dioxide (34, 157, 158, 164, 174–186). Many of those conditions change during tick feeding or differ between tick and vertebrate tissues. Further studies are needed to determine how each of those environmental conditions correlates with the B. burgdorferi infectious cycle. Similarly, it is likely that the consistent infectious cycles of other vector-borne pathogens may also be regulated by detection of physiological changes that occur only at a specific step in the cycle.

## References

[1] Gilbert L. 2021. The impacts of climate change on ticks and tick-borne disease risk. Annu Rev Entomol 66:373–388. 10.1146/annurev-ento-052720-094533. 33417823

[2] Steere AC, Strle F, Wormser GP, Hu LT, Branda JA, Hovius JWR, Li X, Mead PS. 2016. Lyme borreliosis. Nat Rev Dis Primers 2:16090. 10.1038/nrdp.2016.90. 27976670 PMC5539539

[3] Kullberg BJ, Vrijmoeth HD, van de Schoor F, Hovius JW. 2020. Lyme borreliosis: diagnosis and management. BMJ 369:m1041. 10.1136/bmj.m1041. 32457042

[4] Centers for Disease Control and Prevention. 2013. Three sudden cardiac deaths associated with Lyme carditis—United States, November 2012-July 2013. MMWR Morb Mortal Wkly Rep 62:993–996. 24336130 PMC4585587

[5] Radolf JD, Caimano MJ, Stevenson B, Hu LT. 2012. Of ticks, mice, and men: understanding the dual-host lifestyle of Lyme disease spirochaetes. Nat Rev Microbiol 10:87–98. 10.1038/nrmicro2714. 22230951 PMC3313462

[6] Jutras BL, Lochhead RB, Kloos ZA, Biboy J, Strle K, Booth CJ, Govers SK, Gray J, Schumann P, Vollmer W, Bockenstedt LK, Steere AC, Jacobs-Wagner C. 2019. *Borrelia burgdorferi* peptidoglycan is a persistent antigen in patients with Lyme arthritis. Proc Natl Acad Sci USA 116:13498–13507. 10.1073/pnas.1904170116. 31209025 PMC6613144

[7] Anderson C, Brissette CA. 2021. The brilliance of *Borrelia*: mechanisms of host immune evasion by Lyme disease-causing spirochetes. Pathogens 10:281. 10.3390/pathogens10030281. 33801255 PMC8001052

[8] Davis MM, Brock AM, DeHart TG, Boribong BP, Lee K, McClune ME, Chang Y, Cramer N, Liu J, Jones CN, Jutras BL. 2021. The peptidoglycan-associated protein NapA plays an important role in the envelope integrity and in the pathogenesis of the Lyme disease spirochete. PLoS Pathog 17:e1009546. 10.1371/journal.ppat.1009546. 33984073 PMC8118282

[9] Lochhead RB, Strle K, Arvikar SL, Weis JJ, Steere AC. 2021. Lyme arthritis: linking infection, inflammation and autoimmunity. Nat Rev Rheumatol 17:449–461. 10.1038/s41584-021-00648-5. 34226730 PMC9488587

[10] Tilly K, Rosa PA, Stewart PE. 2008. Biology of infection with *Borrelia burgdorferi*. Infect Dis Clin North Am 22:217–234. 10.1016/j.idc.2007.12.013. 18452798 PMC2440571

[11] Stewart PE, Rosa PA. 2018. Physiologic and genetic factors influencing the zoonotic cycle of *Borrelia burgdorferi*. Curr Top Microbiol Immunol 415:63–82. 10.1007/82_2017_43. 28864829

[12] Dunham-Ems SM, Caimano MJ, Pal U, Wolgemuth CW, Eggers CH, Balic A, Radolf JD. 2009. Live imaging reveals a biphasic mode of dissemination of *Borrelia burgdorferi* within ticks. J Clin Invest 119:3652–3665. 10.1172/JCI39401. 19920352 PMC2786795

[13] Fraser CM, Casjens S, Huang WM, Sutton GG, Clayton R, Lathigra R, White O, Ketchum KA, Dodson R, Hickey EK, Gwinn M, Dougherty B, Tomb JF, Fleischmann RD, Richardson D, Peterson J, Kerlavage AR, Quackenbush J, Salzberg S, Hanson M, van Vugt R, Palmer N, Adams MD, Gocayne J, Weidman J, Utterback T, Watthey L, McDonald L, Artiach P, Bowman C, Garland S, Fuji C, Cotton MD, Horst K, Roberts K, Hatch B, Smith HO, Venter JC. 1997. Genomic sequence of a Lyme disease spirochaete, *Borrelia burgdorferi*. Nature 390:580–586. 10.1038/37551. 9403685

[14] Casjens S, Palmer N, van Vugt R, Huang WM, Stevenson B, Rosa P, Lathigra R, Sutton G, Peterson J, Dodson RJ, Haft D, Hickey E, Gwinn M, White O, Fraser C. 2000. A bacterial genome in flux: the twelve linear and nine circular extrachromosomal DNAs of an infectious isolate of the Lyme disease spirochete *Borrelia burgdorferi*. Mol Microbiol 35:490–516. 10.1046/j.1365-2958.2000.01698.x. 10672174

[15] Caimano MJ, Kenedy MR, Kairu T, Desrosiers DC, Harman M, Dunham-Ems S, Akins DR, Pal U, Radolf JD. 2011. The hybrid histidine kinase Hk1 is part of a two-component system that is essential for survival of *Borrelia burgdorferi* in feeding *Ixodes scapularis* ticks. Infect Immun 79:3117–3130. 10.1128/IAI.05136-11. 21606185 PMC3147546

[16] He M, Ouyang Z, Troxell B, Xu H, Moh A, Piesman J, Norgard MV, Gomelsky M, Yang XF. 2011. Cyclic di-GMP is essential for the survival of the Lyme disease spirochete in ticks. PLoS Pathog 7:e1002133. 10.1371/journal.ppat.1002133. 21738477 PMC3128128

[17] Kostick JL, Szkotnicki LT, Rogers EA, Bocci P, Raffaelli N, Marconi RT. 2011. The diguanylate cyclase, Rrp1, regulates critical steps in the enzootic cycle of the Lyme disease spirochetes. Mol Microbiol 81:219–231. 10.1111/j.1365-2958.2011.07687.x. 21542866 PMC3124615

[18] Sze CW, Smith A, Choi YH, Yang X, Pal U, Yu A, Li C. 2013. Study of the response regulator Rrp1 reveals its regulatory role in chitobiose utilization and virulence of *Borrelia burgdorferi*. Infect Immun 81:1775–1787. 10.1128/IAI.00050-13. 23478317 PMC3647990

[19] Bontemps-Gallo S, Lawrence K, Gherardini FC. 2016. Two different virulence-related regulatory pathways in *Borrelia burgdorferi* are directly affected by osmotic fluxes in the blood meal of feeding *Ixodes* ticks. PLoS Pathog 12:e1005791. 10.1371/journal.ppat.1005791. 27525653 PMC4985143

[20] Yang XF, Alani SM, Norgard MV. 2003. The response regulator Rrp2 is essential for the expression of major membrane lipoproteins in *Borrelia burgdorferi*. Proc Natl Acad Sci USA 100:11001–11006. 10.1073/pnas.1834315100. 12949258 PMC196916

[21] Burtnick MN, Downey JS, Brett PJ, Boylan JA, Frye JG, Hoover TR, Gherardini FC. 2007. Insights into the complex regulation of *rpoS* in *Borrelia burgdorferi*. Mol Microbiol 65:277–293. 10.1111/j.1365-2958.2007.05813.x. 17590233 PMC1976401

[22] Boardman BK, He M, Ouyang Z, Xu H, Pang X, Yang XF. 2008. Essential role of the response regulator Rrp2 in the infectious cycle of *Borrelia burgdorferi*. Infect Immun 76:3844–3853. 10.1128/IAI.00467-08. 18573895 PMC2519420

[23] Groshong AM, Gibbons NE, Yang XF, Blevins JS. 2012. Rrp2, a prokaryotic enhancer-like binding protein, is essential for viability of *Borrelia burgdorferi*. J Bacteriol 194:3336–3342. 10.1128/JB.00253-12. 22544267 PMC3434732

[24] Yin Y, Yang Y, Xiang X, Wang Q, Yang ZN, Blevins J, Lou Y, Yang XF. 2016. Insight into the dual functions of bacterial enhancer-binding protein Rrp2 of *Borrelia burgdorferi*. J Bacteriol 198:1543–1552. 10.1128/JB.01010-15. 26977110 PMC4859600

[25] Liu Q, Xu H, Zhang Y, Yang J, Du J, Zhou Y, Yang XF, Lou Y. 2020. Role of HK2 in the enzootic cycle of *Borrelia burgdorferi*. Front Med (Lausanne) 7:573648. 10.3389/fmed.2020.573648. 33195322 PMC7649798

[26] Samuels DS. 2011. Gene regulation in *Borrelia burgdorferi*. Annu Rev Microbiol 65:479–499. 10.1146/annurev.micro.112408.134040. 21801026

[27] Stevenson B, Seshu J. 2018. Regulation of gene and protein expression in the Lyme disease spirochete, p 83–112. *In* Adler B (ed), Spirochete biology: the post genomic era. Springer-Nature, Heidelberg, Germany. 10.1007/82_2017_49 29064060

[28] Samuels DS, Lybecker MC, Yang XF, Ouyang Z, Bourret TJ, Boyle WK, Stevenson B, Drecktrah D, Caimano MJ. 2021. Gene regulation and transcriptomics, p 87–129. *In* Radolf JD, Samuels DS (ed), Lyme disease and relapsing fever spirochetes. Caister Academic Press, London, England. 10.21775/cimb.042.223 PMC7946783 33300497

[29] Jutras BL, Chenail AM, Stevenson B. 2013. Changes in bacterial growth rate govern expression of the *Borrelia burgdorferi* OspC and Erp infection-associated surface proteins. J Bacteriol 195:757–764. 10.1128/JB.01956-12. 23222718 PMC3562092

[30] Barbour AG. 1984. Isolation and cultivation of Lyme disease spirochetes. Yale J Biol Med 57:521–525. 6393604 PMC2589996

[31] Zückert WR. 2007. Laboratory maintenance of *Borrelia burgdorferi*. Curr Protoc Microbiol Chapter 12:Unit 12C.1. 10.1002/9780471729259.mc12c01s4. 18770608

[32] Schwan TG, Piesman J, Golde WT, Dolan MC, Rosa PA. 1995. Induction of an outer surface protein on *Borrelia burgdorferi* during tick feeding. Proc Natl Acad Sci USA 92:2909–2913. 10.1073/pnas.92.7.2909. 7708747 PMC42328

[33] Stevenson B, Schwan TG, Rosa PA. 1995. Temperature-related differential expression of antigens in the Lyme disease spirochete, *Borrelia burgdorferi*. Infect Immun 63:4535–4539. 10.1128/iai.63.11.4535-4539.1995. 7591099 PMC173648

[34] Carroll JA, Garon CF, Schwan TG. 1999. Effects of environmental pH on membrane proteins in *Borrelia burgdorferi*. Infect Immun 67:3181–3187. 10.1128/IAI.67.7.3181-3187.1999. 10377088 PMC116493

[35] von Lackum K, Stevenson B. 2005. Carbohydrate utilization by the Lyme borreliosis spirochete, *Borrelia burgdorferi*. FEMS Microbiol Lett 243:173–179. 10.1016/j.femsle.2004.12.002. 15668016

[36] Kornblatt AN, Steere AC, Brownstein DG. 1984. Experimental Lyme disease in rabbits: spirochetes found in erythema migrans and blood. Infect Immun 46:220–223. 10.1128/iai.46.1.220-223.1984. 6480108 PMC261456

[37] Barthold SW, Moody KD, Terwilliger GA, Duray PH, Jacoby RO, Steere AC. 1988. Experimental Lyme arthritis in rats infected with *Borrelia burgdorferi*. J Infect Dis 157:842–846. 10.1093/infdis/157.4.842. 3258003

[38] Barthold SW, Beck DS, Hansen GM, Terwilliger GA, Moody KD. 1990. Lyme borreliosis in selected strains and ages of laboratory mice. J Infect Dis 162:133–138. 10.1093/infdis/162.1.133. 2141344

[39] Philipp MT, Aydintug MK, Bohm RP, Jr, Cogswell FB, Dennis VA, Lanners HN, Lowrie RC, Roberts ED, Conway MD, Karacorlu M, Peyman GA, Gubler DJ, Johnson BJB, Piesman J, Gu Y. 1993. Early and early disseminated phases of Lyme disease in the rhesus monkey: a model for infection in humans. Infect Immun 61:3047–3059. 10.1128/iai.61.7.3047-3059.1993. 8514412 PMC280958

[40] Woese CR. 1987. Bacterial evolution. Microbiol Rev 51:221–271. 10.1128/mr.51.2.221-271.1987. 2439888 PMC373105

[41] Paster BJ, Dewhirst FE, Weisburg WG, Tordoff LA, Fraser GJ, Hespell RB, Stanton TB, Zablen L, Mandelco L, Woese CR. 1991. Phylogenetic analysis of the spirochetes. J Bacteriol 173:6101–6109. 10.1128/jb.173.19.6101-6109.1991. 1917844 PMC208357

[42] Stevenson B, Bono JL, Elias A, Tilly K, Rosa P. 1998. Transformation of the Lyme disease spirochete *Borrelia burgdorferi* with heterologous DNA. J Bacteriol 180:4850–4855. 10.1128/JB.180.18.4850-4855.1998. 9733687 PMC107509

[43] Rosa PA, Jewett MW. 2021. Genetic manipulation of *Borrelia*. Curr Issues Mol Biol 42:307–332. 10.21775/cimb.042.307. 33300496 PMC7946753

[44] Samuels DS, Marconi RT, Huang WM, Garon CF. 1994. *gyrB* mutations in coumermycin A_1_-resistant *Borrelia burgdorferi*. J Bacteriol 176:3072–3075. 10.1128/jb.176.10.3072-3075.1994. 8188609 PMC205466

[45] Samuels DS, Mach KE, Garon CF. 1994. Genetic transformation of the Lyme disease agent *Borrelia burgdorferi* with coumarin-resistant *gyrB*. J Bacteriol 176:6045–6049. 10.1128/jb.176.19.6045-6049.1994. 7928965 PMC196823

[46] Samuels DS. 1995. Electrotransformation of the spirochete *Borrelia burgdorferi*. Methods Mol Biol 47:253–259. 10.1385/0-89603-310-4:253. 7550741 PMC5815860

[47] Tilly K, Casjens S, Stevenson B, Bono JL, Samuels DS, Hogan D, Rosa P. 1997. The *Borrelia burgdorferi* circular plasmid cp26: conservation of plasmid structure and targeted inactivation of the *ospC* gene. Mol Microbiol 25:361–373. 10.1046/j.1365-2958.1997.4711838.x. 9282748

[48] Bono JL, Elias AF, Kupko JJ, Stevenson B, Tilly K, Rosa P. 2000. Efficient targeted mutagenesis in *Borrelia burgdorferi*. J Bacteriol 182:2445–2452. 10.1128/JB.182.9.2445-2452.2000. 10762244 PMC111306

[49] Stewart PE, Thalken R, Bono JL, Rosa P. 2001. Isolation of a circular plasmid region sufficient for autonomous replication and transformation of infectious *Borrelia burgdorferi*. Mol Microbiol 39:714–721. 10.1046/j.1365-2958.2001.02256.x. 11169111

[50] Eggers CH, Caimano MJ, Clawson ML, Miller WG, Samuels DS, Radolf JD. 2002. Identification of loci critical for replication and compatibility of a *Borrelia burgdorferi* cp32 plasmid and use of a cp32-based shuttle vector for the expression of fluorescent reporters in the Lyme disease spirochaete. Mol Microbiol 43:281–295. 10.1046/j.1365-2958.2002.02758.x. 11985709

[51] Stewart PE, Chaconas G, Rosa P. 2003. Conservation of plasmid maintenance functions between linear and circular plasmids in *Borrelia burgdorferi*. J Bacteriol 185:3202–3209. 10.1128/JB.185.10.3202-3209.2003. 12730180 PMC154063

[52] Stewart PE, Hoff J, Fischer E, Krum JG, Rosa PA. 2004. Genome-wide transposon mutagenesis of *Borrelia burgdorferi* for identification of phenotypic mutants. Appl Environ Microbiol 70:5973–5979. 10.1128/AEM.70.10.5973-5979.2004. 15466540 PMC522107

[53] Blevins JS, Revel AT, Smith AH, Bachlani GN, Norgard MV. 2007. Adaptation of a luciferase gene reporter and *lac* expression system to *Borrelia burgdorferi*. Appl Environ Microbiol 73:1501–1513. 10.1128/AEM.02454-06. 17220265 PMC1828772

[54] Gilbert MA, Morton EA, Bundle SF, Samuels DS. 2007. Artificial regulation of *ospC* expression in *Borrelia burgdorferi*. Mol Microbiol 63:1259–1273. 10.1111/j.1365-2958.2007.05593.x. 17257307

[55] Whetstine CR, Slusser JG, Zückert WR. 2009. Development of a single-plasmid-based regulatable gene expression system for *Borrelia burgdorferi*. Appl Environ Microbiol 75:6553–6558. 10.1128/AEM.02825-08. 19700541 PMC2765148

[56] Hayes BM, Jewett MW, Rosa PA. 2010. *lacZ* reporter system for use in *Borrelia burgdorferi*. Appl Environ Microbiol 76:7407–7412. 10.1128/AEM.01389-10. 20851957 PMC2976203

[57] Kasumba IN, Bestor A, Tilly K, Rosa PA. 2015. Use of an endogenous plasmid locus for stable in *trans* complementation in *Borrelia burgdorferi*. Appl Environ Microbiol 81:1038–1046. 10.1128/AEM.03657-14. 25452278 PMC4292500

[58] Takacs CN, Kloos ZA, Scott M, Rosa PA, Jacobs-Wagner C. 2018. Fluorescent proteins, promoters, and selectable markers for applications in the Lyme disease spirochete *Borrelia burgdorferi*. Appl Environ Microbiol 84:e01824-18. 10.1128/AEM.01824-18. 30315081 PMC6275353

[59] Latham JI, Blevins JS. 2018. Generation of conditional mutants in *Borrelia burgdorferi*. Methods Mol Biol 1690:225–239. 10.1007/978-1-4939-7383-5_17. 29032548

[60] Hillman C, Stewart PE, Strnad M, Stone H, Starr T, Carmody A, Evans TJ, Carracoi V, Wachter J, Rosa PA. 2019. Visualization of spirochetes by labeling membrane proteins with fluorescent biarsenical dyes. Front Cell Infect Microbiol 9:287. 10.3389/fcimb.2019.00287. 31482073 PMC6710359

[61] Takacs CN, Scott M, Chang Y, Kloos ZA, Irnov I, Rosa PA, Liu J, Jacobs-Wagner C. 2021. A CRISPR interference platform for selective downregulation of gene expression in *Borrelia burgdorferi*. Appl Environ Microbiol 87:e02519-20. 10.1128/AEM.02519-20. PMC7851697 33257311

[62] Purser JE, Norris SJ. 2000. Correlation between plasmid content and infectivity in *Borrelia burgdorferi*. Proc Natl Acad Sci USA 97:13865–13870. 10.1073/pnas.97.25.13865. 11106398 PMC17667

[63] Labandeira-Rey M, Seshu J, Skare JT. 2003. The absence of linear plasmid 25 or 28-1 of *Borrelia burgdorferi* dramatically alters the kinetics of experimental infection via distinct mechanisms. Infect Immun 71:4608–4613. 10.1128/IAI.71.8.4608-4613.2003. 12874340 PMC166013

[64] Casjens SR, Gilcrease EB, Vujadinovic M, Mongodin EF, Luft BJ, Schutzer SE, Fraser CM, Qiu WG. 2017. Plasmid diversity and phylogenetic consistency in the Lyme disease agent *Borrelia burgdorferi*. BMC Genomics 18:165. 10.1186/s12864-017-3553-5. 28201991 PMC5310021

[65] Bunikis I, Kutschan-Bunikis S, Bonde M, Bergström S. 2011. Multiplex PCR as a tool for validating plasmid content of *Borrelia burgdorferi*. J Microbiol Methods 86:243–247. 10.1016/j.mimet.2011.05.004. 21605603

[66] Babb K, McAlister JD, Miller JC, Stevenson B. 2004. Molecular characterization of *Borrelia burgdorferi erp* promoter/operator elements. J Bacteriol 186:2745–2756. 10.1128/JB.186.9.2745-2756.2004. 15090516 PMC387816

[67] Schulze RJ, Zückert WR. 2006. *Borrelia burgdorferi* lipoproteins are secreted to the outer surface by default. Mol Microbiol 59:1473–1484. 10.1111/j.1365-2958.2006.05039.x. 16468989

[68] Burgdorfer W, Barbour AG, Hayes SF, Benach JL, Grunwaldt E, Davis JP. 1982. Lyme disease—a tick-borne spirochetosis? Science 216:1317–1319. 10.1126/science.7043737. 7043737

[69] Johnson RC, Schmid GP, Hyde FW, Steigerwalt AG, Brenner DJ. 1984. *Borrelia burgdorferi* sp. nov.: etiologic agent of Lyme disease. Int J Syst Bacteriol 34:496–497. 10.1099/00207713-34-4-496.

[70] Grimm D, Tilly K, Byram R, Stewart PE, Krum JG, Bueschel DM, Schwan TG, Policastro PF, Elias AF, Rosa PA. 2004. Outer-surface protein C of the Lyme disease spirochete: a protein induced in ticks for infection of mammals. Proc Natl Acad Sci USA 101:3142–3147. 10.1073/pnas.0306845101. 14970347 PMC365757

[71] Pal U, Yang X, Chen M, Bockenstedt LK, Anderson JF, Flavell RA, Norgard MV, Fikrig E. 2004. OspC facilitates *Borrelia burgdorferi* invasion of *Ixodes scapularis* salivary glands. J Clin Invest 113:220–230. 10.1172/JCI19894. 14722614 PMC311436

[72] Lagal V, Portnoï D, Faure G, Postic D, Baranton G. 2006. *Borrelia burgdorferi* sensu stricto invasiveness is correlated with OspC-plasminogen affinity. Microbes Infect 8:645–652. 10.1016/j.micinf.2005.08.017. 16513394

[73] Stewart PE, Wang X, Bueschel DM, Clifton DR, Grimm D, Tilly K, Carroll JA, Weis JJ, Rosa PA. 2006. Delineating the requirement for the *Borrelia burgdorferi* virulence factor OspC in the mammalian host. Infect Immun 74:3547–3553. 10.1128/IAI.00158-06. 16714587 PMC1479289

[74] Tilly K, Krum JG, Bestor A, Jewett MW, Grimm D, Bueschel D, Byram R, Dorward D, Vanraden MJ, Stewart P, Rosa P. 2006. *Borrelia burgdorferi* OspC protein is required exclusively in a crucial early stage of mammalian infection. Infect Immun 74:3554–3564. 10.1128/IAI.01950-05. 16714588 PMC1479285

[75] Tilly K, Bestor A, Jewett MW, Rosa P. 2007. Rapid clearance of Lyme disease spirochetes lacking OspC from skin. Infect Immun 75:1517–1519. 10.1128/IAI.01725-06. 17158906 PMC1828573

[76] Tilly K, Bestor A, Dulebohn DP, Rosa PA. 2009. OspC-independent infection and dissemination by host-adapted *Borrelia burgdorferi*. Infect Immun 77:2672–2682. 10.1128/IAI.01193-08. 19398538 PMC2708573

[77] Antonara S, Ristow P, McCarthy J, Coburn J. 2010. Effect of *Borrelia burgdorferi* OspC at the site of inoculation in mouse skin. Infect Immun 78:4723–4733. 10.1128/IAI.00464-10. 20696825 PMC2976318

[78] Earnhart CG, Leblanc DV, Alix KE, Desrosiers DC, Radolf JD, Marconi RT. 2010. Identification of residues within ligand-binding domain 1 (LBD1) of the *Borrelia burgdorferi* OspC protein required for function in the mammalian environment. Mol Microbiol 76:393–408. 10.1111/j.1365-2958.2010.07103.x. 20199597 PMC2917209

[79] Önder Ö, Humphrey PT, McOmber B, Korobova F, Francella N, Greenbaum DC, Brisson D. 2012. OspC is potent plasminogen receptor on surface of *Borrelia burgdorferi*. J Biol Chem 287:16860–16868. 10.1074/jbc.M111.290775. 22433849 PMC3351304

[80] Tilly K, Bestor A, Rosa PA. 2013. Lipoprotein succession in *Borrelia burgdorferi*: similar but distinct roles for OspC and VlsE at different stages of mammalian infection. Mol Microbiol 89:216–227. 10.1111/mmi.12271. 23692497 PMC3713631

[81] Skare JT, Shaw DK, Trzeciakowski JP, Hyde JA. 2016. In vivo imaging demonstrates that *Borrelia burgdorferi ospC* is uniquely expressed temporally and spatially throughout experimental infection. PLoS One 11:e0162501. 10.1371/journal.pone.0162501. 27611840 PMC5017786

[82] Bölin I, Portnoy DA, Wolf-Watz H. 1985. Expression of the temperature-inducible outer membrane proteins of yersiniae. Infect Immun 48:234–240. 10.1128/iai.48.1.234-240.1985. 3980086 PMC261940

[83] Konkel ME, Tilly K. 2000. Temperature-regulated expression of bacterial virulence genes. Microbes Infect 2:157–166. 10.1016/s1286-4579(00)00272-0. 10742688

[84] Akins DR, Porcella SF, Popova TG, Shevchenko D, Baker SI, Li M, Norgard MV, Radolf JD. 1995. Evidence for in vivo but not in vitro expression of a *Borrelia burgdorferi* outer surface protein F (OspF) homologue. Mol Microbiol 18:507–520. 10.1111/j.1365-2958.1995.mmi_18030507.x. 8748034

[85] Stevenson B, Tilly K, Rosa PA. 1996. A family of genes located on four separate 32-kilobase circular plasmids in *Borrelia burgdorferi* B31. J Bacteriol 178:3508–3516. 10.1128/jb.178.12.3508-3516.1996. 8655548 PMC178120

[86] Casjens S, van Vugt R, Tilly K, Rosa PA, Stevenson B. 1997. Homology throughout the multiple 32-kilobase circular plasmids present in Lyme disease spirochetes. J Bacteriol 179:217–227. 10.1128/jb.179.1.217-227.1997. 8982001 PMC178682

[87] Stevenson B, Casjens S, van Vugt R, Porcella SF, Tilly K, Bono JL, Rosa P. 1997. Characterization of cp18, a naturally truncated member of the cp32 family of *Borrelia burgdorferi* plasmids. J Bacteriol 179:4285–4291. 10.1128/jb.179.13.4285-4291.1997. 9209045 PMC179251

[88] Caimano MJ, Yang X, Popova TG, Clawson ML, Akins DR, Norgard MV, Radolf JD. 2000. Molecular and evolutionary characterization of the cp32/18 family of supercoiled plasmids in *Borrelia burgdorferi* 297. Infect Immun 68:1574–1586. 10.1128/IAI.68.3.1574-1586.2000. 10678977 PMC97318

[89] Stevenson B, Zückert WR, Akins DR. 2001. Repetition, conservation, and variation: the multiple cp32 plasmids of *Borrelia* species, p 87–100. *In* Saier MH, García-Lara J (ed), The spirochetes: molecular and cellular biology. Horizon Press, Oxford, United Kingdom.

[90] Casjens SR, Eggers CH, Schwartz I. 2010. *Borrelia* genomics: chromosome, plasmids, bacteriophages and genetic variation, p 27–54. *In* Samuels DS, Radolf JD (ed), *Borrelia*: molecular biology, host interaction and pathogenesis. Caister Academic Press, Norfolk, United Kingdom.

[91] Brisson D, Zhou W, Jutras BL, Casjens S, Stevenson B. 2013. Distribution of cp32 prophages among Lyme disease-causing spirochetes and natural diversity of their lipoprotein-encoding *erp* loci. Appl Environ Microbiol 79:4115–4128. 10.1128/AEM.00817-13. 23624478 PMC3697573

[92] Schwartz I, Margos G, Casjens SR, Qiu WG, Eggers CH. 2021. Multipartite genome of Lyme disease *Borrelia*: structure, variation, and prophages, p 17–62. *In* Radolf JD, Samuels DS (ed), Lyme disease and relapsing fever spirochetes. Caister Academic Press, London, United Kingdom.

[93] Stevenson B, Bykowski T, Cooley AE, Babb K, Miller JC, Woodman ME, von Lackum K, Riley SP. 2006. The Lyme disease spirochete Erp lipoprotein family: structure, function and regulation of expression, p 354–372. *In* Cabello FC, Godfrey HP, Hulinska D (ed), Molecular biology of spirochetes. IOS Press, Amsterdam, The Netherlands.

[94] Lam TT, Nguyen T-PK, Montgomery RR, Kantor FS, Fikrig E, Flavell RA. 1994. Outer surface proteins E and F of *Borrelia burgdorferi*, the agent of Lyme disease. Infect Immun 62:290–298. 10.1128/iai.62.1.290-298.1994. 8262642 PMC186099

[95] El-Hage N, Babb K, Carroll JA, Lindstrom N, Fischer ER, Miller JC, Gilmore RD, Mbow ML, Stevenson B. 2001. Surface exposure and protease insensitivity of *Borrelia burgdorferi* Erp (OspEF-related) lipoproteins. Microbiology (Reading) 147:821–830. 10.1099/00221287-147-4-821. 11283278

[96] El-Hage N, Stevenson B. 2002. Simultaneous coexpression of *Borrelia burgdorferi* Erp proteins occurs through a specific, *erp* locus-directed regulatory mechanism. J Bacteriol 184:4536–4543. 10.1128/JB.184.16.4536-4543.2002. 12142424 PMC135249

[97] Nguyen T-PK, Lam TT, Barthold SW, Telford SR, Flavell RA, Fikrig E. 1994. Partial destruction of *Borrelia burgdorferi* within ticks that engorged on OspE- or OspF-immunized mice. Infect Immun 62:2079–2084. 10.1128/iai.62.5.2079-2084.1994. 8168973 PMC186469

[98] Miller JC, von Lackum K, Babb K, McAlister JD, Stevenson B. 2003. Temporal analysis of *Borrelia burgdorferi* Erp protein expression throughout the mammal-tick infectious cycle. Infect Immun 71:6943–6952. 10.1128/IAI.71.12.6943-6952.2003. 14638783 PMC308935

[99] Hefty PS, Brooks CS, Jett AM, White GL, Wikel SK, Kennedy RC, Akins DR. 2002. OspE-related, OspF-related, and Elp lipoproteins are immunogenic in baboons experimentally infected with *Borrelia burgdorferi* and in human Lyme disease patients. J Clin Microbiol 40:4256–4265. 10.1128/JCM.40.11.4256-4265.2002. 12409407 PMC139709

[100] Hellwage J, Meri T, Heikkilä T, Alitalo A, Panelius J, Lahdenne P, Seppälä IJT, Meri S. 2001. The complement regulatory factor H binds to the surface protein OspE of *Borrelia burgdorferi*. J Biol Chem 276:8427–8435. 10.1074/jbc.M007994200. 11113124

[101] Brissette CA, Haupt K, Barthel D, Cooley AE, Bowman A, Skerka C, Wallich R, Zipfel PF, Kraiczy P, Stevenson B. 2009. *Borrelia burgdorferi* infection-associated surface proteins ErpP, ErpA, and ErpC bind human plasminogen. Infect Immun 77:300–306. 10.1128/IAI.01133-08. 19001079 PMC2612283

[102] Brissette CA, Verma A, Bowman A, Cooley AE, Stevenson B. 2009. The *Borrelia burgdorferi* outer-surface protein ErpX binds mammalian laminin. Microbiology (Reading) 155:863–872. 10.1099/mic.0.024604-0. 19246757 PMC10010501

[103] Lin Y-P, Bhowmick R, Coburn J, Leong JM. 2015. Host cell heparan sulfate glycosaminoglycans are ligands for OspF-related proteins of the Lyme disease spirochete. Cell Microbiol 17:1464–1476. 10.1111/cmi.12448. 25864455 PMC4583806

[104] Pereira MJ, Wager B, Garrigues RJ, Gerlach E, Quinn JD, Dowdell A, Osburne MS, Zückert WR, Kraiczy P, Garcia BL, Leong JM. 2021. Lipoproteome screening of the Lyme disease agent identifies novel inhibitors of antibody-mediated complement killing. bioRxiv. 10.1101/2021.09.23.461563. PMC9060444 35312359

[105] El-Hage N, Lieto LD, Stevenson B. 1999. Stability of *erp* loci during *Borrelia burgdorferi* infection: recombination is not required for chronic infection of immunocompetent mice. Infect Immun 67:3146–3150. 10.1128/IAI.67.6.3146-3150.1999. 10338534 PMC96635

[106] Stevenson B. 2002. *Borrelia burgdorferi erp* (*ospE*-related) gene sequences remain stable during mammalian infection. Infect Immun 70:5307–5311. 10.1128/IAI.70.9.5307-5311.2002. 12183589 PMC128278

[107] Sung SY, McDowell JV, Carlyon JA, Marconi RT. 2000. Mutation and recombination in the upstream homology box-flanked *ospE*-related genes of the Lyme disease spirochetes result in the development of new antigenic variants during infection. Infect Immun 68:1319–1327. 10.1128/IAI.68.3.1319-1327.2000. 10678944 PMC97285

[108] Goethert HK, Mather TN, Buchthal J, Telford SR. 2021. Retrotransposon-based blood meal analysis of nymphal deer ticks demonstrates spatiotemporal diversity of *Borrelia burgdorferi* and *Babesia microti* reservoirs. Appl Environ Microbiol 87:e02370-20. 10.1128/AEM.02370-20. 33158895 PMC7783337

[109] Stevenson B, El-Hage N, Hines MA, Miller JC, Babb K. 2002. Differential binding of host complement inhibitor factor H by *Borrelia burgdorferi* Erp surface proteins: a possible mechanism underlying the expansive host range of Lyme disease spirochetes. Infect Immun 70:491–497. 10.1128/IAI.70.2.491-497.2002. 11796574 PMC127719

[110] McDowell JV, Wolfgang J, Tran E, Metts MS, Hamilton D, Marconi RT. 2003. Comprehensive analysis of the factor H binding capabilities of *Borrelia* species associated with Lyme disease: delineation of two distinct classes of factor H binding proteins. Infect Immun 71:3597–3602. 10.1128/IAI.71.6.3597-3602.2003. 12761145 PMC155754

[111] Porcella SF, Popova TG, Akins DR, Li M, Radolf JD, Norgard MV. 1996. *Borrelia burgdorferi* supercoiled plasmids encode multi-copy tandem open reading frames and a lipoprotein gene family. J Bacteriol 178:3293–3307. 10.1128/jb.178.11.3293-3307.1996. 8655511 PMC178083

[112] Porcella SF, Fitzpatrick CA, Bono JL. 2000. Expression and immunological analysis of the plasmid-borne *mlp* genes of *Borrelia burgdorferi* strain B31. Infect Immun 68:4992–5001. 10.1128/IAI.68.9.4992-5001.2000. 10948116 PMC101720

[113] Carroll JA, El-Hage N, Miller JC, Babb K, Stevenson B. 2001. *Borrelia burgdorferi* RevA antigen is a surface-exposed outer membrane protein whose expression is regulated in response to environmental temperature and pH. Infect Immun 69:5286–5293. 10.1128/IAI.69.9.5286-5293.2001. 11500397 PMC98637

[114] Yang XF, Hübner A, Popova TG, Hagman KE, Norgard MV. 2003. Regulation of expression of the paralogous Mlp family in *Borrelia burgdorferi*. Infect Immun 71:5012–5020. 10.1128/IAI.71.9.5012-5020.2003. 12933844 PMC187337

[115] Bykowski T, Woodman ME, Cooley AE, Brissette CA, Brade V, Wallich R, Kraiczy P, Stevenson B. 2007. Coordinated expression of *Borrelia burgdorferi* complement regulator-acquiring surface proteins during the Lyme disease spirochete’s mammal-tick infection cycle. Infect Immun 75:4227–4236. 10.1128/IAI.00604-07. 17562769 PMC1951152

[116] Brissette CA, Bykowski T, Cooley AE, Bowman A, Stevenson B. 2009. *Borrelia burgdorferi* RevA antigen binds host fibronectin. Infect Immun 77:2802–2812. 10.1128/IAI.00227-09. 19398540 PMC2708576

[117] Hammerschmidt C, Koenigs A, Siegel C, Hallström T, Skerka C, Wallich R, Zipfel PF, Kraiczy P. 2014. Versatile roles of CspA orthologs in complement inactivation of serum-resistant Lyme disease spirochetes. Infect Immun 82:380–392. 10.1128/IAI.01094-13. 24191298 PMC3911871

[118] Hart TM, Dupuis AP, Tufts DM, Blom AM, Starkey SR, Rego ROM, Ram S, Kraiczy P, Kramer LD, Diuk-Wasser MA, Kolokotronis S-O, Lin Y-P. 2021. Host tropism determination by convergent evolution of immunological evasion in the Lyme disease system. PLoS Pathog 17:e1009801. 10.1371/journal.ppat.1009801. 34324600 PMC8354441

[119] Hefty PS, Jolliff SE, Caimano MJ, Wikel SK, Radolf JD, Akins DR. 2001. Regulation of OspE-related, OspF-related, and Elp lipoproteins of *Borrelia burgdorferi* strain 297 by mammalian host-specific signals. Infect Immun 69:3618–3627. 10.1128/IAI.69.6.3618-3627.2001. 11349022 PMC98350

[120] Babb K, Bykowski T, Riley SP, Miller MC, DeMoll E, Stevenson B. 2006. *Borrelia burgdorferi* EbfC, a novel, chromosomally-encoded protein, binds specific DNA sequences adjacent to *erp* loci on the spirochete’s resident cp32 prophages. J Bacteriol 188:4331–4339. 10.1128/JB.00005-06. 16740939 PMC1482946

[121] Jutras BL, Verma A, Stevenson B. 2012. Identification of novel DNA-binding proteins using DNA-affinity chromatography/pull down. Curr Protoc Microbiol Chapter 1:Unit1.F1. 10.1002/9780471729259.mc01f01s24. PMC3564586 22307548

[122] Burns LH, Adams CA, Riley SP, Jutras BL, Bowman A, Chenail AM, Cooley AE, Haselhorst LA, Moore AM, Babb K, Fried MG, Stevenson B. 2010. BpaB, a novel protein encoded by the Lyme disease spirochete’s cp32 prophages, binds to *erp* Operator 2 DNA. Nucleic Acids Res 38:5443–5455. 10.1093/nar/gkq284. 20421207 PMC2938228

[123] Jutras BL, Chenail AM, Carroll DW, Miller MC, Zhu H, Bowman A, Stevenson B. 2013. Bpur, the Lyme disease spirochete's PUR-domain protein: identification as a transcriptional modulator and characterization of nucleic acid interactions. J Biol Chem 288:26220–26234. 10.1074/jbc.M113.491357. 23846702 PMC3764826

[124] Stevenson B, Casjens S, Rosa P. 1998. Evidence of past recombination events among the genes encoding the Erp antigens of *Borrelia burgdorferi*. Microbiology 144:1869–1879. 10.1099/00221287-144-7-1869. 9695920

[125] Zückert WR, Meyer J. 1996. Circular and linear plasmids of Lyme disease spirochetes have extensive homology: characterization of a repeated DNA element. J Bacteriol 178:2287–2298. 10.1128/jb.178.8.2287-2298.1996. 8636030 PMC177937

[126] Jutras BL, Verma A, Adams CA, Brissette CA, Burns LH, Whetstine CR, Bowman A, Chenail AM, Zückert WR, Stevenson B. 2012. BpaB and EbfC DNA-binding proteins regulate production of the Lyme disease spirochete’s infection-associated Erp surface proteins. J Bacteriol 194:778–786. 10.1128/JB.06394-11. 22155777 PMC3272957

[127] Jutras BL, Savage CR, Arnold WK, Lethbridge KG, Carroll DW, Tilly K, Bestor A, Zhu H, Seshu J, Zückert WR, Stewart PE, Rosa PA, Brissette CA, Stevenson B. 2019. The Lyme disease spirochete’s BpuR DNA/RNA-binding protein is differentially expressed during the mammal-tick infectious cycle, which affects translation of the SodA superoxide dismutase. Mol Microbiol 112:973–991. 10.1111/mmi.14336. 31240776 PMC6736767

[128] Graebsch A, Roche S, Kostrewa D, Söding J, Niessing D. 2010. Of bits and bugs—on the use of bioinformatics and a bacterial crystal structure to solve a eukaryotic repeat-protein structure. PLoS One 5:e13402. 10.1371/journal.pone.0013402. 20976240 PMC2954813

[129] Daniel DC, Johnson EM. 2018. PURA, the gene encoding Pur-alpha, member of an ancient nucleic acid-binding protein family with mammalian neurological functions. Gene 643:133–143. 10.1016/j.gene.2017.12.004. 29221753 PMC5770235

[130] Jutras BL, Jones G, Verma A, Brown NA, Antonicello AD, Chenail AM, Stevenson B. 2013. Post-transcriptional autoregulation of the Lyme disease bacterium's BpuR DNA/RNA-binding protein. J Bacteriol 195:4915–4923. 10.1128/JB.00819-13. 23974034 PMC3807498

[131] Riley SP, Bykowski T, Cooley AE, Burns LH, Babb K, Brissette CA, Bowman A, Rotondi M, Miller MC, DeMoll E, Lim K, Fried MG, Stevenson B. 2009. *Borrelia burgdorferi* EbfC defines a newly-identified, widespread family of bacterial DNA-binding proteins. Nucleic Acids Res 37:1973–1983. 10.1093/nar/gkp027. 19208644 PMC2665219

[132] Flower AM, McHenry CS. 1991. Transcriptional organization of the *Escherichia coli dnaX* gene. J Mol Biol 220:649–658. 10.1016/0022-2836(91)90107-H. 1870125

[133] Lim K, Tempczyk A, Parsons JF, Bonander N, Toedt J, Kelman Z, Howard A, Eisenstein E, Herzberg O. 2003. Crystal structure of YbaB from *Haemophilus influenzae* (HI0442), a protein of unknown function coexpressed with the recombinational DNA repair protein RecR. Proteins 50:375–379. 10.1002/prot.10297. 12486730

[134] Cooley AE, Riley SP, Kral K, Miller MC, DeMoll E, Fried MG, Stevenson B. 2009. DNA-binding by *Haemophilus influenzae* and *Escherichia coli* YbaB, members of a widely-distributed bacterial protein family. BMC Microbiol 9:137. 10.1186/1471-2180-9-137. 19594923 PMC2720974

[135] Wang H, Wang F, Hua X, Ma T, Chen J, Xu X, Wang L, Tian B, Hua Y. 2012. Genetic and biochemical characteristics of the histone-like protein DR0199 in *Deinococcus radiodurans*. Microbiology (Reading) 158:936–943. 10.1099/mic.0.053702-0. 22282513

[136] Perez-Rueda E, Ibarra JA. 2015. Distribution of putative xenogeneic silencers in prokaryote genomes. Comput Biol Chem 58:167–172. 10.1016/j.compbiolchem.2015.06.007. 26247404

[137] Pal P, Modi M, Ravichandran S, Yennamalli RM, Priyadarshini R. 2021. DNA-binding properties of YbaB, a putative nucleoid-associated protein from *Caulobacter crescentus*. Front Microbiol 12:733344. 10.3389/fmicb.2021.733344. 34777284 PMC8581549

[138] Jutras BL, Bowman A, Brissette CA, Adams CA, Verma A, Chenail AM, Stevenson B. 2012. EbfC (YbaB) is a new type of bacterial nucleoid-associated protein, and a global regulator of gene expression in the Lyme disease spirochete. J Bacteriol 194:3395–3406. 10.1128/JB.00252-12. 22544270 PMC3434759

[139] Chenail AM, Jutras BL, Adams CA, Burns LH, Bowman A, Verma A, Stevenson B. 2012. *Borrelia burgdorferi* cp32 BpaB modulates expression of the prophage NucP nuclease and SsbP single-stranded DNA-binding protein. J Bacteriol 194:4570–4578. 10.1128/JB.00661-12. 22730122 PMC3415502

[140] Flower AM, McHenry CS. 1990. The γ subunit of DNA polymerase III holoenzyme of *Escherichia coli* is produced by ribosomal frameshifting. Proc Natl Acad Sci USA 87:3713–3717. 10.1073/pnas.87.10.3713. 2187190 PMC53973

[141] Chen K, Saxena P, Walker JR. 1993. Expression of the *Escherichia coli dnaX* gene. J Bacteriol 175:6663–6670. 10.1128/jb.175.20.6663-6670.1993. 7691799 PMC206778

[142] Winzeler E, Shapiro L. 1996. A novel promoter motif for *Caulobacter* cell cycle-controlled DNA replication genes. J Mol Biol 264:412–425. 10.1006/jmbi.1996.0650. 8969294

[143] Keiler KC, Shapiro L. 2001. Conserved promoter motif is required for cell cycle timing of *dnaX* transcription in *Caulobacter*. J Bacteriol 183:4860–4865. 10.1128/JB.183.16.4860-4865.2001. 11466289 PMC99540

[144] Caimano MJ, Iyer R, Eggers CH, Gonzalez C, Morton EA, Gilbert MA, Schwartz I, Radolf JD. 2007. Analysis of the RpoS regulon in *Borrelia burgdorferi* in response to mammalian host signals provides insight into RpoS function during the enzootic cycle. Mol Microbiol 65:1193–1217. 10.1111/j.1365-2958.2007.05860.x. 17645733 PMC2967192

[145] Hübner A, Yang X, Nolen DM, Popova TG, Cabello PC, Norgard MV. 2001. Expression of *Borrelia burgdorferi* OspC and DbpA is controlled by a RpoN-RpoS regulatory pathway. Proc Natl Acad Sci USA 98:12724–12729. 10.1073/pnas.231442498. 11675503 PMC60121

[146] Yang XF, Lybecker MC, Pal U, Alani SM, Blevins J, Revel AT, Samuels DS, Norgard MV. 2005. Analysis of the *ospC* regulatory element controlled by the RpoN-RpoS regulatory pathway in *Borrelia burgdorferi*. J Bacteriol 187:4822–4829. 10.1128/JB.187.14.4822-4829.2005. 15995197 PMC1169512

[147] Caimano MJ, Groshong AM, Belperron A, Mao J, Hawley KL, Luthra A, Graham DE, Earnhart CG, Marconi RT, Bockenstedt LK, Blevins JS, Radolf JD. 2019. The RpoS gatekeeper in *Borrelia burgdorferi*: an invariant regulatory scheme that promotes spirochete persistence in reservoir hosts and niche diversity. Front Microbiol 10:1923. 10.3389/fmicb.2019.01923. 31507550 PMC6719511

[148] Xu Q, McShan K, Liang FT. 2007. Identification of an *ospC* operator critical for immune evasion of *Borrelia burgdorferi*. Mol Microbiol 64:220–231. 10.1111/j.1365-2958.2007.05636.x. 17376084

[149] Xu Q, McShan K, Liang FT. 2008. Verification and dissection of the *ospC* operator by using *flaB* promoter as a reporter in *Borrelia burgdorferi*. Microb Pathog 45:70–78. 10.1016/j.micpath.2008.03.002. 18479884 PMC2497006

[150] Drecktrah D, Hall LS, Hoon-Hanks LL, Samuels DS. 2013. An inverted repeat in the ospC operator is required for induction in *Borrelia burgdorferi*. PLoS One 8:e68799. 10.1371/journal.pone.0068799. 23844242 PMC3700930

[151] Dunham-Ems SM, Caimano MJ, Eggers CH, Radolf JD. 2012. *Borrelia burgdorferi* requires the alternative sigma factor RpoS for dissemination within the vector during tick-to-mammal transmission. PLoS Pathog 8:e1002532. 10.1371/journal.ppat.1002532. 22359504 PMC3280991

[152] Smith AH, Blevins JS, Bachlani GN, Yang XF, Norgard MV. 2007. Evidence that RpoS (σ^S^) in *Borrelia burgdorferi* is controlled directly by RpoN (σ^54^/σ^N^). J Bacteriol 189:2139–2144. 10.1128/JB.01653-06. 17158681 PMC1855718

[153] Ouyang Z, Blevins JS, Norgard MV. 2008. Transcriptional interplay among the regulators Rrp2, RpoN and RpoS in *Borrelia burgdorferi*. Microbiology (Reading) 154:2641–2658. 10.1099/mic.0.2008/019992-0. 18757798

[154] Blevins JS, Xu H, He M, Norgard MV, Reitzer L, Yang XF. 2009. Rrp2, a sigma54-dependent transcriptional activator of *Borrelia burgdorferi*, activates *rpoS* in an enhancer-independent manner. J Bacteriol 191:2902–2905. 10.1128/JB.01721-08. 19201806 PMC2668385

[155] Lybecker MC, Samuels DS. 2007. Temperature-induced regulation of RpoS by a small RNA in *Borrelia burgdorferi*. Mol Microbiol 64:1075–1089. 10.1111/j.1365-2958.2007.05716.x. 17501929

[156] Lybecker MC, Abel CA, Feig AL, Samuels DS. 2010. Identification and function of the RNA chaperone Hfq in the Lyme disease spirochete *Borrelia burgdorferi*. Mol Microbiol 78:622–635. 10.1111/j.1365-2958.2010.07374.x. 20815822 PMC2963666

[157] Boylan JA, Posey JE, Gherardini FC. 2003. *Borrelia* oxidative stress response regulator, BosR: a distinct Zn-dependent transcriptional activator. Proc Natl Acad Sci USA 100:11684–11689. 10.1073/pnas.2032956100. 12975527 PMC208818

[158] Hyde JA, Shaw DK, Smith R, Trzeciakowski JP, Skare JT. 2009. The BosR regulatory protein of *Borrelia burgdorferi* interfaces with the RpoS regulatory pathway and modulates both the oxidative stress response and pathogenic properties of the Lyme disease spirochete. Mol Microbiol 74:1344–1355. 10.1111/j.1365-2958.2009.06951.x. 19906179 PMC2805275

[159] Samuels DS, Radolf JD. 2009. Who is the BosR around here anyway? Mol Microbiol 74:1295–1299. 10.1111/j.1365-2958.2009.06971.x. 19943896 PMC3005592

[160] Ouyang Z, Deka RK, Norgard MV. 2011. BosR (BB0647) controls the RpoN-RpoS regulatory pathway and virulence expression in *Borrelia burgdorferi* by a novel DNA-binding mechanism. PLoS Pathog 7:e1001272. 10.1371/journal.ppat.1001272. 21347346 PMC3037356

[161] Miller CL, Karna SL, Seshu J. 2013. Borrelia host adaptation regulator (BadR) regulates *rpoS* to modulate host adaptation and virulence factors in *Borrelia burgdorferi*. Mol Microbiol 88:105–124. 10.1111/mmi.12171. 23387366 PMC4828661

[162] Ouyang Z, Zhou J, Brautigam CA, Deka RK, Norgard MV. 2014. Identification of a core sequence for the binding of BosR to the *rpoS* promoter region in *Borrelia burgdorferi*. Microbiology (Reading) 160:851–862. 10.1099/mic.0.075655-0. 24608174 PMC3992719

[163] Ouyang Z, Zhou J. 2015. BadR (BB0693) controls growth phase-dependent induction of *rpoS* and *bosR* in *Borrelia burgdorferi* via recognizing TAAAATAT motifs. Mol Microbiol 98:1147–1167. 10.1111/mmi.13206. 26331438

[164] Wang P, Yu Z, Santangelo TJ, Olesik J, Wang Y, Heldwein E, Li X. 2017. BosR is a novel Fur family member responsive to copper and regulating copper homeostasis in *Borrelia burgdorferi*. J Bacteriol 199:e00276-17. 10.1128/JB.00276-17. 28583949 PMC5527378

[165] Sarkar A, Hayes BM, Dulebohn DP, Rosa PA. 2011. Regulation of the virulence determinant OspC by bbd18 on linear plasmid lp17 of *Borrelia burgdorferi*. J Bacteriol 193:5365–5373. 10.1128/JB.01496-10. 21784941 PMC3187453

[166] Casselli T, Tourand Y, Bankhead T. 2012. Altered murine tissue colonization by *Borrelia burgdorferi* following targeted deletion of linear plasmid 17-carried genes. Infect Immun 80:1773–1782. 10.1128/IAI.05984-11. 22354033 PMC3347435

[167] Dulebohn DP, Hayes BM, Rosa PA. 2014. Global repression of host-associated genes of the Lyme disease spirochete through post-transcriptional modulation of the alternative sigma factor RpoS. PLoS One 9:e93141. 10.1371/journal.pone.0093141. 24671196 PMC3966842

[168] Hayes BM, Dulebohn DP, Sarkar A, Tilly K, Bestor A, Ambroggio X, Rosa PA. 2014. Regulatory protein BBD18 of the Lyme disease spirochete: essential role during tick acquisition? mBio 5:e01017-14. 10.1128/mBio.01017-14. PMC3977360 24692636

[169] Ouyang Z, Narasimhan S, Neelakanta G, Kumar M, Pal U, Fikrig E, Norgard MV. 2012. Activation of the RpoN-RpoS regulatory pathway during the enzootic life cycle of *Borrelia burgdorferi*. BMC Microbiol 12:44. 10.1186/1471-2180-12-44. 22443136 PMC3320556

[170] Piesman J, Oliver JR, Sinsky RJ. 1990. Growth kinetics of the Lyme disease spirochete (*Borrelia burgdorferi*) in vector ticks (*Ixodes dammini*). Am J Trop Med Hyg 42:352–357. 10.4269/ajtmh.1990.42.352. 2331043

[171] de Silva AM, Fikrig E. 1995. Growth and migration of *Borrelia burgdorferi* in *Ixodes* ticks during blood feeding. Am J Trop Med Hyg 53:397–404. 10.4269/ajtmh.1995.53.397. 7485694

[172] Piesman J, Schneider BS, Zeidner NS. 2001. Use of quantitative PCR to measure density of *Borrelia burgdorferi* in the midgut and salivary glands of feeding tick vectors. J Clin Microbiol 39:4145–4148. 10.1128/JCM.39.11.4145-4148.2001. 11682544 PMC88501

[173] Piesman J, Schneider BS. 2002. Dynamic changes in Lyme disease spirochetes during transmission by nymphal ticks. Exp Appl Acarol 28:141–145. 10.1023/a:1025351727785. 14570124

[174] Hyde JA, Seshu J, Skare JT. 2006. Transcriptional profiling of *Borrelia burgdorferi* containing a unique *bosR* allele identifies a putative stress regulon. Microbiology (Reading) 152:2599–2609. 10.1099/mic.0.28996-0. 16946255

[175] Hyde JA, Trzeciakowski JP, Skare JT. 2007. *Borrelia burgdorferi* alters its gene expression and antigenic profile in response to CO_2_ levels. J Bacteriol 189:437–445. 10.1128/JB.01109-06. 17098904 PMC1797391

[176] Karna SLR, Sanjuan E, Esteve-Gassent MD, Miller CL, Maruskova M, Seshu J. 2011. CsrA modulates levels of lipoproteins and key regulators of gene expression critical for pathogenic mechianisms of *Borrelia burgdorferi*. Infect Immun 79:732–744. 10.1128/IAI.00882-10. 21078860 PMC3028860

[177] Troxell B, Ye M, Yang Y, Carrasco SE, Lou Y, Yang XF. 2013. Manganese and zinc regulate virulence determinants in *Borrelia burgdorferi*. Infect Immun 81:2743–2752. 10.1128/IAI.00507-13. 23690398 PMC3719580

[178] Drecktrah D, Lybecker M, Popitsch N, Rescheneder P, Hall LS, Samuels DS. 2015. The *Borrelia burgdorferi* RelA/SpoT homolog and stringent response regulate survival in the tick vector and global gene expression during starvation. PLoS Pathog 11:e1005160. 10.1371/journal.ppat.1005160. 26371761 PMC4570706

[179] Khajanchi BK, Odeh E, Gao L, Jacobs MB, Philipp MT, Lin T, Norris SJ. 2015. Phosphoenolpyruvate phosphotransferase system components modulate gene transcription and virulence of Borrelia burgdorferi. Infect Immun 84:754–764. 10.1128/IAI.00917-15. 26712207 PMC4771366

[180] Caimano MJ, Drecktrah D, Kung F, Samuels DS. 2016. Interaction of the Lyme disease spirochete with its tick vector. Cell Microbiol 18:919–927. 10.1111/cmi.12609. 27147446 PMC5067140

[181] Dulebohn DP, Richards CL, Su H, Lawrence KA, Gherardini FC. 2017. Weak organic acids decrease *Borrelia burgdorferi* cytoplasmic pH, eliciting an acid stress response and impacting RpoN- and RpoS-dependent gene expression. Front Microbiol 8:1734. 10.3389/fmicb.2017.01734. 29033900 PMC5626856

[182] Lin YH, Romo JA, Smith TC, Reyes AN, Karna SL, Miller CL, Van Laar TA, Yendapally R, Chambers JP, Seshu J. 2017. Spermine and spermidine alter gene expression and antigenic profile of *Borrelia burgdorferi*. Infect Immun 85:e00684-16. 10.1128/IAI.00684-16. 28052993 PMC5328495

[183] Zhang JJ, Chen T, Yang Y, Du J, Li H, Troxell B, He M, Carrasco SE, Gomelsky M, Yang XF. 2018. Positive and negative regulation of glycerol utilization by the c-di-GMP binding protein PlzA in *Borrelia burgdorferi*. J Bacteriol 200:e00243-18. 10.1128/JB.00243-18. 30181123 PMC6199477

[184] Lin YH, Chen Y, Smith TC, Karna SLR, Seshu J. 2018. Short-chain fatty acids alter metabolic and virulence attributes of *Borrelia burgdorferi*. Infect Immun 86:e00217-18. 10.1128/IAI.00217-18. 29891543 PMC6105876

[185] Yang X, Goldberg MS, Popova TG, Schoeler GB, Wikel SK, Hagman KE, Norgard MV. 2000. Interdependence of environmental factors influencing reciprocal patterns of gene expression in virulent *Borrelia burgdorferi*. Mol Microbiol 37:1470–1479. 10.1046/j.1365-2958.2000.02104.x. 10998177

[186] DeHart TG, Kushelman MR, Hildreth SB, Helm RF, Jutras BL. 2021. The unusual cell wall of the Lyme disease spirochaete *Borrelia burgdorferi* is shaped by a tick sugar. Nat Microbiol 6:1583–1592. 10.1038/s41564-021-01003-w. 34819646 PMC8612929

